# Gender differences in outcome and use of resources do exist in Swedish intensive care, but to no advantage for women of premenopausal age

**DOI:** 10.1186/s13054-015-0873-1

**Published:** 2015-03-30

**Authors:** Carolina Samuelsson, Folke Sjöberg, Göran Karlström, Thomas Nolin, Sten M Walther

**Affiliations:** Department of Intensive and Perioperative Care, Skåne University Hospital, Malmö and Lund, Inga Marie Nilssons gata 47 pl. 3, SE21428, Malmö, Sweden; Department of Anesthesiology and Intensive Care, Halland Hospital, Halmstad, Sweden; Department of Clinical and Experimental Medicine, Faculty of Health Sciences, Linköping University, Linköping, Sweden; Burn Center, Department of Hand, Plastic Surgery and Intensive Care, Linköping University Hospital, Linköping County Council, Linköping, Sweden; The Swedish Intensive Care Registry, Karlstad, Sweden; Department of Anesthesiology and Intensive Care, Kristianstad Central Hospital, Kristianstad, Sweden; Division of Cardiovascular Medicine, Department of Medical and Health Sciences, Linköping University, Linköping, Sweden

## Abstract

**Introduction:**

Preclinical data indicate that oestrogen appears to play a beneficial role in the pathophysiology of and recovery from critical illness. In few previous epidemiologic studies, however, have researchers analysed premenopausal women as a separate group when addressing potential gender differences in critical care outcome. Our aim was to see if women of premenopausal age have a better outcome following critical care and to investigate the association between gender and use of intensive care unit (ICU) resources.

**Methods:**

On the basis of our analysis of 127,254 consecutive Simplified Acute Physiology Score III–scored Swedish Intensive Care Registry ICU admissions from 2008 through 2012, we determined the risk-adjusted 30-day mortality, accumulated nurse workload score and ICU length of stay. To investigate associations with sex, we used logistic regression and multivariate analyses on the entire cohort as well as on two subgroups stratified by median age for menopause (up to and including 45 years and older than 45 years) and six selected diagnostic subgroups (sepsis, multiple trauma, chronic obstructive pulmonary disease, acute respiratory distress syndrome, pneumonia and cardiac arrest).

**Results:**

There was no sex difference in risk-adjusted mortality for the cohort as a whole, and there was no sex difference in risk-adjusted mortality in the group 45 years of age and younger. For the group of patients older than 45 years of age, we found a reduced risk-adjusted mortality in men admitted for cardiac arrest. For the cohort as a whole, and for those admitted with multiple trauma, male sex was associated with a higher nurse workload score and a longer ICU stay.

**Conclusions:**

Using information derived from a large multiple ICU register database, we found that premenopausal female sex was not associated with a survival advantage following intensive care in Sweden. When the data were adjusted for age and severity of illness, we found that men used more ICU resources per admission than women did.

**Electronic supplementary material:**

The online version of this article (doi:10.1186/s13054-015-0873-1) contains supplementary material, which is available to authorized users.

## Introduction

There are substantial preclinical data on how male and female sex hormones play a role in the pathophysiology of and recovery from critical illness. Oestrogen is a potent antioxidant that appears to have a protective effect in trauma and haemorrhage [1-3], whereas high testosterone levels in critical conditions are associated with suppression of cardiac function and the immunologic response [4-7].

Despite the apparent advantage of females observed in the experimental setting, findings in epidemiologic critical care studies have been equivocal; risk-adjusted gender differences in mortality are reported to be disparate, potentially diagnosis-specific [8-11] or due to differences in treatment and resource allocation [12-14]. It was also recently shown that increasing blood oestrogen levels during ongoing intensive care are associated with mortality in both sexes, but it is unknown whether this association is causative or consequential [15]. If higher initial physiologic baseline oestrogen levels are beneficial in critical illness, one should expect premenopausal women to have a superior outcome. Indeed, some studies indicate that women of premenopausal age have higher intensive care unit (ICU) survival [16], a more beneficial physiologic response to severe trauma [17] and better outcome following cardiac arrest as compared to other demographic groups [18,19]. Other studies, however, have failed to demonstrate such an advantage [12,20].

More than 90% of all Swedish ICUs currently report their admissions to the Swedish Intensive Care Registry (SIR), a nationwide comparative audit database for intensive care. To describe case mix, enable comparisons and audit outcomes, the SIR uses the Simplified Acute Physiology Score III (SAPS III), which is one of the three major groups of prediction models currently used in general intensive care [21,22]. The SAPS III score is derived from data on the patient’s comorbidity, location before admission, hospital stay prior to intensive care admission, reason(s) for ICU admission, surgical status, presence of infection and degree of physiologic derangement ±1 hour from ICU admission. The compound SAPS III score can also be transformed into a probability called the estimated mortality risk (EMR), which is the predicted risk of death before being discharged from the hospital [23].

The Nursing Care Recording System (NCR11) is the major nurse workload score used by Swedish ICUs to describe use of resources and ICU occupancy, and most ICUs record and report it to the SIR [24]. A strength of the NCR11 score is that it describes the nursing component of patient care and components related to medical procedures. Nurse workload scores have previously been used to measure and compare resource allocation between sexes, with varying results [25-27].

The primary aim of the present study was to evaluate the association between sex and 30-day mortality in a Swedish general ICU cohort in which the study population was dichotomized at the median age for female menopause (45 years) to see if a premenopausal female hormone profile is associated with better outcome following intensive care. In addition to looking at the entire unselected general intensive care cohort, we also wanted to apply this question in six more well-defined diagnostic subgroups. We also set out to investigate the association of sex with use of intensive care resources.

## Methods

Swedish ICUs began reporting ICU admissions according to the SAPS III guidelines in 2008. In this retrospective study, we analysed all 127,254 SAPS III–scored adult ICU patients (>15 years of age) who had validated mortality outcome data in the SIR and whose ICU admission occurred between 1 January 2008 and 31 December 2012. Sixty-five ICUs reported to the SIR during this period. In the registry, an ICU admission is defined as an admission to the ICU of a patient whose condition is acute (life-threatening) and who requires monitoring, diagnostic examinations and/or treatment. Admissions due to hospital bed shortage or for routine postoperative recovery were not considered ICU admissions for the purposes of the present study.

To describe illness severity at admission, the EMR was derived from the SAPS III score according to the general SAPS III equation (logit = −32.6659 + ln(SAPS III score +20.5958) × 7.3068; EMR = elogit/(1 + elogit)) [28]. When determining the SAPS III score, the SAPS III model requires each patient to be assigned one or several reasons for his or her ICU admission from among a predefined set [28]. At ICU discharge, each patient was assigned a primary ICU diagnosis from among a predefined list of diagnoses (from the International Statistical Classification of Diseases and Related Health Problems, Tenth Revision (ICD-10)) provided by the SIR. An admission could thus have several SAPS III ‘admission reasons’, but always only one primary ICU ICD-10 diagnosis. The primary diagnosis was used to define six major diagnostic subgroups (sepsis, multiple trauma, cardiac arrest, chronic obstructive pulmonary disease (COPD) exacerbation, acute respiratory distress syndrome (ARDS) and pneumonia). Sepsis, multiple trauma and cardiac arrest were included because previous experimental data indicate that sex hormone levels may influence the pathophysiology of these conditions [29,30]. For COPD, ARDS and pneumonia, previously published experimental and epidemiologic data are less supportive of a sex difference [8,31-33].

In addition to SAPS III variables, the SIR also contains extensive demographic, administrative and outcome data for each ICU admission. Through automatic cross-checking between the SIR and the Swedish National Death Register using the unique Swedish personal identity number, the SIR contains reliable 30-day and 90-day mortality data for 98% of all registered ICU admissions. Two reasons for not being able to obtain these mortality data are non-Swedish residency and patients with concealed identity.

The NCR11 score has similarities with the Nursing Activity Score [34], the Therapeutic Intervention Scoring System and the Nine Equivalents of Nurse Manpower Use Score [24]. Briefly, the NCR11 system categorizes intensive care measures and procedures into 11 categories related to monitoring and treatment of different organ systems, invasive procedures, care of wounds and/or drains and support of patients’ next of kin. Depending on time taken and/or occurrence of a procedure, each of the categories is assigned a score from 0 to 3 points. A value of 0 points is given if no procedure or measure within the category is performed during that nursing shift. At the end of each nursing shift, the scores are added and a total score out of 33 is given. The length of ICU admission (hours) and the accumulated NCR11 score (points) for each admission were used in this study as a proxy for use of resources.

### Ethics and consent

The SIR is a prospectively collected nationwide database of individual intensive care patient records, and it operates within the legal framework of all Swedish national quality registers. In accordance with this legal framework written informed consent was not sought and not required from each individual patient. All patients or their next of kin were however informed of the collection of data and had the opportunity to not contribute with data to the registry. The data from the SIR used in this study had all personal identifiers removed. The present study was conducted in compliance with the Declaration of Helsinki and was approved by the regional ethics review board of Linköping University as well as by the SIR research steering committee.

### Statistical analysis

Age, EMR, total NCR11 score and ICU length of stay (LOS) had non-Gaussian distributions; median values are therefore presented for these variables. Differences were analysed using the Mann–Whitney *U* test. Sex differences in mortality and SAPS III admission reasons were examined with the Pearson’s χ^2^ test.

For our primary analysis of the entire study cohort, multivariate logistic regression was used to assess associations between sex and the dependent variable 30-day mortality, controlling for potential confounders. We included variables describing patient age and comorbidity (comorbid chronic illnesses as defined and scored in the SAPS III model [35]), hospital LOS in days (after logarithmic transformation), location before ICU admission, major therapeutic options used before ICU admission (as defined in the SAPS III model), reason for ICU admission (as defined and scored in the SAPS III model), surgical status and presence of an infection in the model. The ICU admission physiologic derangement score (in theory from 0 to 77 points, identical to box III of the SAPS III model [35]) was also included. The variables that were included in the main model of the primary analysis of the entire study cohort were then similarly used to assess the associations between sex and 30-day mortality in the age subgroups and in the diagnostic subgroups. Model fit is presented as the c-statistic and McFadden’s *R*^2^.

To assess the association of sex with use of ICU resources, when controlling for age and severity of illness, we developed two multivariate linear regression models with sex, SAPS III EMR and age as explanatory variables. The first model had length of ICU stay (ICU LOS) as the dependent outcome variable and was applied to the entire cohort (n =127,254). The second model had the accumulated NCR11 score for each admission as the dependent outcome variable and was applied to the NCR11-scored admissions (n =87,646). Estimated β-coefficients for male sex are presented in the Results section below, and sex was regarded as being associated with resource use if the estimated β-coefficients had a *P*-value below the significance level. Two-sided *P*-values (when <0.05) and 95% or 99% confidence intervals (CIs) are presented in the Results section. Where hypotheses were tested using multiple sex comparisons in three age groups and six diagnostic subgroups, an adjusted and conservative significance level at *P* <0.001 was chosen.

STATA/SE 13.1 (StataCorp, College Station, TX, USA) and STATISTICA 64 (StatSoft, Tulsa, OK, USA) software were used for data handling and statistical analyses.

## Results

ICU admission characteristics, demographics and outcomes are presented in Table 1 for the study cohort as a whole and for males and females. The most common SAPS III admission reasons were respiratory, neurological and cardiovascular. Median values in the entire study cohort were 64 years for age, 21% for EMR, 24 hours for ICU LOS, 56 points for NCR11 score upon admission, 8.2% for ICU mortality, 18% for 30-day mortality and 22% for 90-day mortality.

**Table 1:** **Description of the study groups with respect to Simplified Acute Physiology Score III admission characteristics, demographics and outcomes** ^**a**^

		**All admissions**	**Male**	**Female**	***P*** **-value** ^**b**^
Number of admissions (%)		127,254	72,295 (57 %)	54,959 (43 %)	
Median age, yr (IQR)		64 (45 to 75)	64 (47 to 74)	63 (43 to 75)	
Median SAPS III score (IQR)		52 (41 to 64)	53 (42 to 65)	52 (41 to 63)	<0.001
Median SAPS III EMR (IQR)		0.21 (0.07 to 0.44)	0.22 (0.08 to 0.46)	0.21 (0.07 to 0.42)	<0.001
Median length of ICU stay, hr (IQR)		24 (12.7 to 60.6)	24.8 (12.7 to 65.3)	23.3 (12.6 to 53.4)	<0.001
Median NCR11 score (IQR)		56 (30 to 127)	58 (30 to 137)	53 (29 to 115)	<0.001
ICU mortality, % (95% CI)		8.2 (8.1 to 8.4)	8.4 (8.2 to 8.6)	7.9 (7.7 to 8.1)	<0.001
30-day mortality, % (95% CI)		17.7 (17.5 to 17.9)	18.0 (17.7 to 18.3)	17.2 (16.9 to 17.5)	<0.001
90-day mortality, % (95% CI)		21.8 (21.6 to 22.0)	22.3 (22.0 to 22.6)	21.3 (21.0 to 21.6)	<0.001
Proportion of cohort with SAPS III reason for admission,^c^ %					
	Basic and observation	15	14	16	<0.001
	Cardiovascular	33	34	32	<0.001
	Hepatic	19	18	20	0.001
	Gastrointestinal	29	29	30	<0.001
	Neurologic	48	47	49	0.04
	Renal	27	28	27	<0.001
	Respiratory	49	48	50	
	Hematologic	20	19	21	
	Metabolic	32	30	35	<0.001
	Trauma	25	26	22	<0.001
	Other	25	24	27	<0.001

^a^CI, Confidence interval; EMR, Estimated mortality risk; ICU, Intensive care unit; IQR, Interquartile range; NCR11, Nursing Care Recording System; SAPS III, Simplified Acute Physiology Score III. ^b^ *P*-value for sex difference. ^c^An admission can be assigned several SAPS III reasons for admission to ICU.

Our primary 30-day mortality logistic regression model analysis is presented in Table 2 for the entire study cohort and per age group. There was no sex difference in 30-day mortality for the entire study cohort (female:male odds ratio (OR), 1.03; 99% CI, 0.98 to 1.08). There was also no difference in the group aged ≤45 years (female:male OR, 0.97; 99% CI, 0.80 to 1.18) or in the group aged >46 years (female:male OR, 1.03; 99% CI, 0.98 to 1.07).

**Table 2:** **Multivariate logistic regression analysis of 30-day mortality for the entire study cohort and age groups** ^**a**^

	**All admissions**	**Age ≤45 yr**	**Age >45 yr**
	**(n =127,254)**	**(n =31,939)**	**(n =95,315)**
Male sex	Reference	Reference	Reference
Female sex	1.03 (0.98 to 1.08)	0.97 (0.80 to 1.18)	1.03 (0.98 to 1.07)
Comorbidity^b^	1.07 (1.06 to 1.07)	1.14 (1.11 to 1.18)	1.06 (1.06 to 1.07)
Age, per yr	1.06 (1.05 to 1.06)	1.03 (1.02 to 1.04)	1.06 (1.06 to 1.06)
Days in hospital prior to ICU^c^	1.23 (1.20 to 1.26)	1.25 (1.13 to 1.39)	1.22 (1.19 to 1.26)
Location prior to ICU admission			
Emergency room	Reference	Reference	Reference
Other ICU	1.15 (1.04 to 1.28)	1.45 (0.97 to 2.15)	1.14 (1.02 to 1.27)
Operating theatre	0.81 (0.72 to 0.92)	0.99 (0.56 to 1.72)	0.80 (0.71 to 0.91)
Recovery room	1.18 (0.87 to 1.60)	0.82 (0.06 to 11.92)	1.17 (0.86 to 1.59)
High-dependency unit	0.93 (0.77 to 1.11)	0.60 (0.19 to 1.84)	0.94 (0.78 to 1.13)
Any other location	1.18 (1.11 to 1.25)	2.00 (1.54 to 2.62)	1.14 (1.02 to 1.27)
Therapy prior to ICU admission^d^	1.22 (1.14 to 1.32)	1.85 (1.38 to 2.50)	1.19 (1.10 to 1.28)
Reasonss for ICU admission, per point^b^	1.07 (1.07 to 1.08)	1.10 (1.07 to 1.12)	1.07 (1.07 to 1.08)
Planned admission	0.91 (0.83 to 1.00)	1.02 (0.66 to 1.57)	0.91 (0.82 to 1.00)
Surgical status			
Emergency surgery	Reference	Reference	Reference
Elective surgery	0.49 (0.43 to 0.55)	0.57 (0.27 to 1.21)	0.49 (0.43 to 0.55)
No surgery	1.35 (1.24 to 1.46)	2.27 (1.52 to 3.41)	1.32 (1.21 to 1.43)
Presence of nosocomial infection	1.07 (0.96 to 1.18)	0.79 (0.50 to 1.24)	1.08 (0.97 to 1.20)
Presence of lower-airway infection	0.75 (0.70 to 0.79)	0.79 (0.60 to 1.03)	0.75 (0.70 to 0.80)
Physiologic derangement, per point^b^	1.10 (1.09 to 1.10)	1.13 (1.12 to 1.14)	1.09 (1.09 to 1.10)
Tertiary care hospital	Reference	Reference	Reference
District general hospital	1.08 (1.02 to 1.14)	0.80 (0.64 to 0.99)	1.10 (1.04 to 1.17)
Local hospital	1.00 (0.93 to 1.07)	0.62 (0.46 to 0.85)	1.02 (0.96 to 1.10)
Model fit			
c-statistic	0.85	0.91	0.81
*R* ^2^	0.28	0.34	0.22

^a^ICU, Intensive care unit. Odds ratios (99% confidence intervals) are presented for all variables in the main 30-day mortality logistic regression model; c-statistics and *R* ^2^-values are presented for assessment of model fit. ^b^Scored as in the Simplified Acute Physiology Score III (SAPS III) model. ^c^Transformed with use of ln(*x*). ^d^Defined as in the SAPS III model.

Thirty-day mortality ORs for sex in the diagnostic subgroups are presented in Table 3. Male sex was associated with a lower mortality rate in patients with cardiac arrest (female:male OR, 1.34; 99% CI, 1.11 to 1.63; *P* <0.001).

**Table 3:** **Associations between sex and 30-day mortality in six major diagnoses using the main multivariate model** ^**a**^

**Diagnosis and age group**	**Female:male**	***P*** **-value**	**c-statistic**	***R*** ^**2**^ **-value**
	**OR (99% CI)**			
Cardiac arrest admissions (n =4,249)	1.34 (1.11 to 1.63)	<0.001	0.74	0.14
≤45 yr (n =344)	1.47 (0.75 to 2.89)		0.76	0.16
>45 yr (n =3,905)	1.33 (1.08 to 1.63)	<0.001	0.75	0.14
ARDS admissions (n =564)	0.73 (0.43 to 1.23)		0.76	0.16
≤45 yr (n =108)	0.85 (0.14 to 5.21)		0.85	0.31
>45 yr (n =456)	0.65 (0.36 to 1.18)		0.76	0.16
Sepsis admissions (n =9,830)	1.17 (1.03 to 1.33)	0.002	0.77	0.16
≤45 yr (n =1,008)	1.08 (0.56 to 2.09)		0.85	0.26
v>45 yr (n =8,822)	1.17 (1.03 to 1.34)	0.002	0.75	0.14
Multiple trauma admissions (n =4,320)	1.09 (0.70 to 1.69)		0.92	0.40
≤45 yr (n =2,148)	0.66 (0.23 to 1.93)		0.94	0.47
>45 yr (n =2,172)	1.0 (0.60 to 1.65)		0.90	0.38
Pneumonia admissions (n =4,448)	0.88 (0.72 to 1.07)		0.76	0.15
≤45 yr (n =580)	1.80 (0.57 to 5.68)		0.91	0.35
>45 yr (n =3,868)	0.83 (0.68 to 1.03)	0.024	0.73	0.12
COPD admissions (n =3,191)	0.94 (0.75 to 1.18)		0.69	0.08
≤45 yr (n =21)	Too few observations			
>45 yr (n =3,179)	0.94 (0.75 to 1.18)		0.69	0.08

^a^ARDS, Acute respiratory distress syndrome; COPD, Chronic obstructive pulmonary disease. Odds ratio (OR) with 99% confidence interval (CI) and model fit (c-statistic and *R* ^2^) were calculated per diagnosis group and per age group using the main multivariate logistic regression model. See Methods section and Table 2 for details. *P*-values <0.05 are presented.

When multivariate linear regression was used to assess the association between sex and ICU resource use, male sex was independently associated with longer ICU LOS in the entire study cohort (all ages β =0.05, *P* <0.001; ≤45 years β =0.05, *P* <0.001; >45 years β =0.04, *P* <0.001) as well as in the multiple trauma subgroup (all ages β =0.14, *P* <0.001; ≤45 years β =0.12, *P* =0.01; >45 years β =0.13, *P* =0.003). Similarly, male sex was independently associated with higher NCR11 score in the entire study cohort (all ages β =0.06, *P* <0.001, ≤45 years β =0.05, *P* <0.001; >45 years β =0.04, *P* <0.001) and in the multiple trauma subgroup (all ages β =0.20, *P* <0.001; ≤45 years β =0.28, *P* <0.001; >45 years β =0.13, *P* =0.01).

## Discussion

In this multiple-ICU, nationwide Swedish register study, we investigated outcome and use of resources in critically ill men and women in age groups defined by the median onset of menopause. Female patients aged ≤45 years had no better risk-adjusted 30-day mortality for either the entire cohort or any of the six major diagnostic subgroups investigated. Instead, a lower mortality was associated with male sex for cardiac arrest. Male sex was independently associated with greater use of intensive care resources for the entire cohort and for multiple trauma patients.

Organizational, sociological and biological factors may contribute to sex differences when it comes to need for, use of and outcome after critical care. Fifty-seven percent of our ICU admissions were men, and males were sicker on admission. These observations are in line with previously reported series [9,10,12,17] and it has been suggested that male patients are more likely to be admitted for intensive care and to receive aggressive life support [10,16,36].

The rationale for the expectation that women of childbearing age will have superior outcomes following critical care is that studies in numerous rat models have shown how important oestrogens are in maintaining organ function during critical illness [29,30]. In a small number of studies on human volunteers and critically ill patients, researchers have reported that women show more favourable physiologic and immunologic responses when challenged with sepsis and hypovolemia [1,37,38], thus providing some clinical support for this notion. We observed no improved outcome in women of premenopausal age following critical care. This does not exclude a protective effect of oestrogens, because such an advantage could be counteracted by opposing factors such as differences in management and resource use. Our findings could also be interpreted such that differences in basal oestrogen levels are of less importance in a modern real-life clinical setting where aggressive intensive care resuscitation protocols may diminish any relative advantage of a premenopausal female hormone profile [15]. Furthermore, we do not know to what extent rodent experiments can be applied to humans, because primates, in contrast to rodents, can increase their peripheral, non-gonadal oestrogen production in response to stress [39,40].

Nursing workload scores have previously been used to describe critical care resource use in men and women with severe sepsis [25] and trauma [26]. Men had higher overall NCR11 scores per admission, as well as longer ICU stays, than women did in our cohort. A higher incidence of complications and resource-requiring intensive care conditions in men could possibly explain this difference [41], but it could also be that health care professionals and relatives are influenced by the patient’s sex in their attitudes, approach and expectations regarding care. The NCR11 score includes medical procedures as well as nursing components of patient care, which makes it a feasible proxy for measuring overall ICU resource use. Its limitations and generalizability must be considered however, since a range of different factors all can increase the score; e.g. when the nurse spends a lot of time with the patient’s relatives, when renal replacement therapy is initiated or when the ICU stay is very long.

Positive effects of female sex hormones have particularly been suggested in trauma [29,30], and the results of an ongoing study where male haemorrhaging trauma patients were randomized to oestrogen or placebo in the acute resuscitation phase may soon be available [42]. The composition of our multiple trauma subgroup was similar to previously reported series with respect to age, sex and injury severity [43,44], and we detected no sex difference with regard to risk-adjusted mortality in any of the age groups, which is in line with previous observations [16,45]. Male trauma patients had higher NCR11 scores and longer ICU stays than female patients in our study, and similar observations were recently made in a Brazilian trauma cohort [26]. An increased incidence of complications, such as multiple organ failure and sepsis, in male trauma patients could possibly explain the difference in resource use [41,45], but other studies suggest that patient sex does not influence complication rates following multiple trauma [46]. Nearly three of four trauma patients in our cohort were men, and the fact that severe trauma patients are usually men may provide an alternative, albeit speculative, reason for the observed sex difference in resource use. Clinical skills and therapeutic intuition are partly empirical, and clinicians promptly recognize conditions, events and clinical presentations that they have experienced before [47]. This may lead to a difference in diagnostic and therapeutic interventions in the comparatively rare female trauma group.

Cardiac arrest in men >45 years of age showed the greatest relative survival advantage in our study, and several previous studies have identified an advantage for men with respect to both treatment and outcome in ischaemic heart conditions [48,49]. Being young (<55 years) and being female were associated with improved outcome in a large US cohort of almost 20,000 prehospital cardiac arrests [18], but these results could not be confirmed in a similar Australian study [19]. Compared with these two large cardiac arrest studies in the emergency room (ER) setting, our ICU cardiac arrest population was small, and any subtle sex-associated differences in outcomes of patients younger than 45 years of age would have remained undetected. Furthermore, Swedish cardiac arrest patients are primarily admitted to the ICU for controlled hypothermia treatment when they fail to regain consciousness following resuscitation and return of spontaneous circulation (ROSC). Our ICU cardiac arrest cohort is thus very different from an ER-treated prehospital cardiac arrest population that includes those who regain consciousness as well as those who never have ROSC.

Higher ICU survival in female patients with COPD exacerbation was recently reported in a US cohort [16]. Similarly to a large study by the Intensive Care National Audit and Research Centre (ICNARC) [8], however, we observed no sex-related differences in mortality among patients with ICU-treated COPD exacerbations. The ICNARC study investigators found higher survival rates in female patients with pneumonia [8], but the 30-day survival advantage in older female patients with pneumonia did not reach statistical significance (*P* =0.024) in our present study.

In our study, there was no sex-related difference in outcomes in sepsis patients ≤45 years of age. Sepsis patients >45 years of age had a female:male OR of 1.17 (*P* =0.002), which is not significant, but potentially interesting. Sex steroids do influence the sepsis-induced complex immunological responses that involve cytokines, immune and endothelial cell interactions [50]. Previous epidemiologic sepsis studies have, however, been disparate with respect to survival, and researchers have reported no sex differences [16,51], higher survival rates in men [14,52] or higher survival rates in women >50 years old [25].

We observed no sex differences with respect to use of resources or mortality in those patients with a primary ICU diagnosis of ARDS. Women may have an increased propensity to develop ARDS [53], but previous audits have not identified sex as an independent predictor of outcome [32,33].

We dichotomized our cohort at the age of 45 years, which is the median age of onset of menopause in Scandinavian women [54]. Previously, both 50 years [12,16,25] and 45 years [17,19] have been used to denote the age of onset of menopause. When repeating our analyses with 50 instead of 45 years of age used to represent the onset of menopause, we arrived at similar results (data not shown).

A strength of this study is that we analysed a large, nationwide multicentre cohort for sex differences with respect to 30-day mortality. However, the study’s design and data handling imply limitations that must be considered. We chose to include all SAPS III–scored ICU admissions registered in the SIR during the study period, regardless of ICU LOS, because our aim was to use a cohort representing the entire adult Swedish ICU population. Hospital and ICU admissions are relatively short in Scandinavia [55], presumably reflecting a low number of hospital and ICU beds per capita [56], a high standard of care and the provision of palliative care outside the ICU setting. Half of the admissions in our cohort lasted less than 24 hours in duration, and this may have distorted any sex differences present in those receiving critical care for a longer period of time.

It should also be noted that the definition of the diagnostic subgroups in this study is strict. All SIR-reporting ICUs have access to software-integrated guidelines on how to assign ICD-10 diagnoses and how to score according to the SAPS III system. When defining the subgroups, we used only the assigned primary ICU ICD-10 diagnosis. Each admission could therefore never belong to more than one diagnostic subgroup, even if clinical criteria were met for more than one of the six selected diagnoses. For conditions such as cardiac arrest, sepsis and multiple trauma, the choice of a primary diagnosis is generally evident, but there are invariably cases where the selection of the primary ICD-10 diagnosis can be difficult, such as when a patient with COPD is admitted to the ICU in respiratory failure and diagnosed with pneumonia.

## Conclusions

In this study, we found no evidence that women of premenopausal age have lower risk-adjusted mortality than men and postmenopausal women in a general non-selected nationwide ICU cohort, nor did we find such evidence in six major diagnostic subgroups. In patients older than the median age of menopause onset, we identified sex-associated, risk-adjusted mortality differences in patients admitted due to cardiac arrest, where a male survival advantage was identified. Male sex was further independently associated with receiving more ICU care per admission. A more detailed analysis of admission characteristics, therapeutic interventions and incidence of complications in the diagnostic subgroups is needed to identify factors underlying the differences observed.

## Key messages

- There was no evidence of lower risk-adjusted 30-day mortality in females of premenopausal age in a 4-year, nationwide Swedish ICU cohort.
- There was evidence of reduced risk-adjusted 30-day mortality in males older than 45 years of age admitted for cardiac arrest.
- Male sex was associated with increased ICU resource use.

## Abbreviations

ARDS: Acute respiratory distress syndrome
CI: Confidence interval
COPD: Chronic obstructive pulmonary disease
EMR: Estimated mortality risk
ER: Emergency room
ICD-10: International Statistical Classification of Diseases and Related Health Problems, Tenth Revision
ICNARC: Intensive Care National Audit and Research Centre
ICU: Intensive care unit
LOS: Length of stay
NCR11: Nursing Care Recording System
OR: Odds ratio
ROSC: Return of spontaneous circulation
SIR: Swedish Intensive Care Registry
SAPS III: Simplified Acute Physiology Score III
